# Risk factors and long-term prognostic impact of immune related pancreatic injury in patients receiving immune checkpoint inhibitors

**DOI:** 10.3389/fimmu.2025.1590992

**Published:** 2025-07-29

**Authors:** Yu Akazawa, Takuto Nosaka, Yosuke Murata, Tomoko Tanaka, Kazuto Takahashi, Tatsushi Naito, Masahiro Ohtani, Yasunari Nakamoto

**Affiliations:** Second Department of Internal Medicine, Faculty of Medical Sciences, University of Fukui, Fukui, Japan

**Keywords:** adverse events, immune checkpoint inhibitors, long-term prognosis, pancreatic injury, risk factor

## Abstract

**Background:**

With the widespread use of immune checkpoint inhibitors (ICIs), the management of immune-related adverse events (irAEs) has become increasingly important. ICI-induced pancreatic injury (ICI-PI) is rare, and its clinical characteristics remain unclear. This study aimed to clarify the risk factors for the development of ICI-PI and prognostic impact of ICI-PI.

**Methods:**

A total of 1039 patients with malignant tumors who received ICI therapy were recruited from September 2014 to December 2024 for this retrospective study. The clinical and pathological characteristics of ICI-PI, including risk factors and prognostic impact, were analyzed. The onset of ICI-PI and irAEs of other organs were defined according to CTCAE ver5.0. The pathological characteristics were evaluated using pancreatic tissue specimens obtained by endoscopic ultrasound-guided fine-needle biopsy.

**Results:**

Of the 982 patients (703 males, 279 females; median age, 71.1 years) included in the study, 48 (4.9%) developed ICI-PI (Grades 2, 3, and 4 in 41, 3, and 4 cases, respectively), and 6 patients (0.6%) developed pancreatitis. Multivariate analysis revealed that the high serum amylase levels before ICI administration (odds ratio, 6.10; 95%CI, 2.55-14.6; *P* < 0.001) and the onset of irAE in other organs (odds ratio, 3.49; 95%CI, 1.88-6.49; *P* < 0.001) were independent risk factors for ICI-PI development. The incidence of other organ irAEs was significantly higher in the ICI-PI onset group than in the ICI-PI non-onset group (*P* < 0.001). Additionally, there was significantly better overall survival in the ICI-PI onset group than in the ICI-PI non-onset group (*P* < 0.001), which was corroborated by a landmark analysis. Also, pathological examination of ICI-related pancreatitis using multiplex fluorescence immunohistochemistry demonstrated infiltration of predominantly CD8 positive T lymphocytes contained abundant granzyme B into the pancreatic parenchyma.

**Conclusions:**

High serum amylase levels before ICI administration and development of other organ irAEs were identified as novel risk factors for ICI-PI onset, and the long-term prognosis was better in patients with ICI-PI. This finding suggests that thorough systemic management, including proactive evaluation of serum amylase levels and comprehensive monitoring for various irAEs, can contribute to early detection of ICI-PI, potentially leading to improved patient outcomes.

## 1 Introduction

Malignant tumors are one of the leading causes of death worldwide, with approximately 1.8 million cases of all types of cancer, and 600,000 cancer deaths per year in the United States (1). Recently, advances in the diagnostic and therapeutic methods for various cancers have greatly improved patient prognosis (2). In particular, the advent of immune checkpoint inhibitors (ICIs), including cytotoxic T-lymphocyte antigen-4 (CTLA-4) inhibitors, programmed cell death protein (PD)-1 inhibitors, and PD ligand inhibitors, has revolutionized the field of cancer therapy (3). In fact, numerous clinical trials have confirmed the efficacy of ICI immunotherapy for malignant tumors (4–6). Based on the above evidence, ICIs have emerged as one of the standard therapies for patients with various malignancies, contributing to the rapid increase in their use. By facilitating T-cell activation and proliferation, abrogating T-regulatory cell functions, and boosting human autoimmunity, ICIs eliminate cancer cells through the upregulation of anti-tumor immune activity (7). Therefore, ICIs demonstrate dramatically anti-tumor responses, which are not observed with conventional chemotherapy.

However, excessive activation of the immune system by ICIs commonly results in unique adverse events, termed immune-related adverse events (irAEs), which are unexpected with conventional chemotherapy. irAEs can be observed in any organ system, and range in severity from mild self-limiting symptoms to severe life-threatening events. The most common involved organ systems include the dermatologic, gastrointestinal, hepatic, pulmonary, and endocrine systems (8). On the other hand, ICI-related pancreatic injury (ICI-PI) is very rare (9–11), and its incidence, risk factors, clinical course, and impact on patient prognosis remain unclear.

Previously, several clinical factors, such as sarcopenia (12), female sex (13), history of autoimmune disease (14), and combination therapy (15) have been reported as risk factors for irAE development. However, reliable risk factors, which could completely prevent irAEs, remain to be identified. Moreover, knowledge concerning risk factors for ICI-PI is more limited.

Several studies have reported the association between irAE onset and patient prognosis. Previous systematic reviews and meta-analyses have demonstrated a better prognosis in patients developing irAEs in various organ systems (16–18). This result could be attributed to T cell activation against antigens common to cancer and normal cells; thus, patients who develop irAEs may have a stronger anti-tumor effect on cancer cells (19). On the other hand, several studies reported no correlation between irAE onset and long-term prognosis (20). Therefore, the findings concerning these associations remain inconsistent.

Recently, it has been established that patients with ICI-PI not only present with elevated pancreatic enzyme levels, but also occasionally develop severe acute pancreatitis, which can be life-threatening. Indeed, a recent meta-analysis reported that nearly 60% of ICI-PI cases reach Grade ≥ 3 severity (21). However, current guidelines regarding ICI-PI are based on limited evidence and warrant revision (22). In the present study, we aimed to clarify the clinical characteristics, including the risk factors for ICI-PI onset and the impact of ICI-PI on long-term prognosis, in patients with advanced cancer who receiving ICIs.

## 2 Materials and methods

### 2.1 Patients and study design

**Figure 1:**
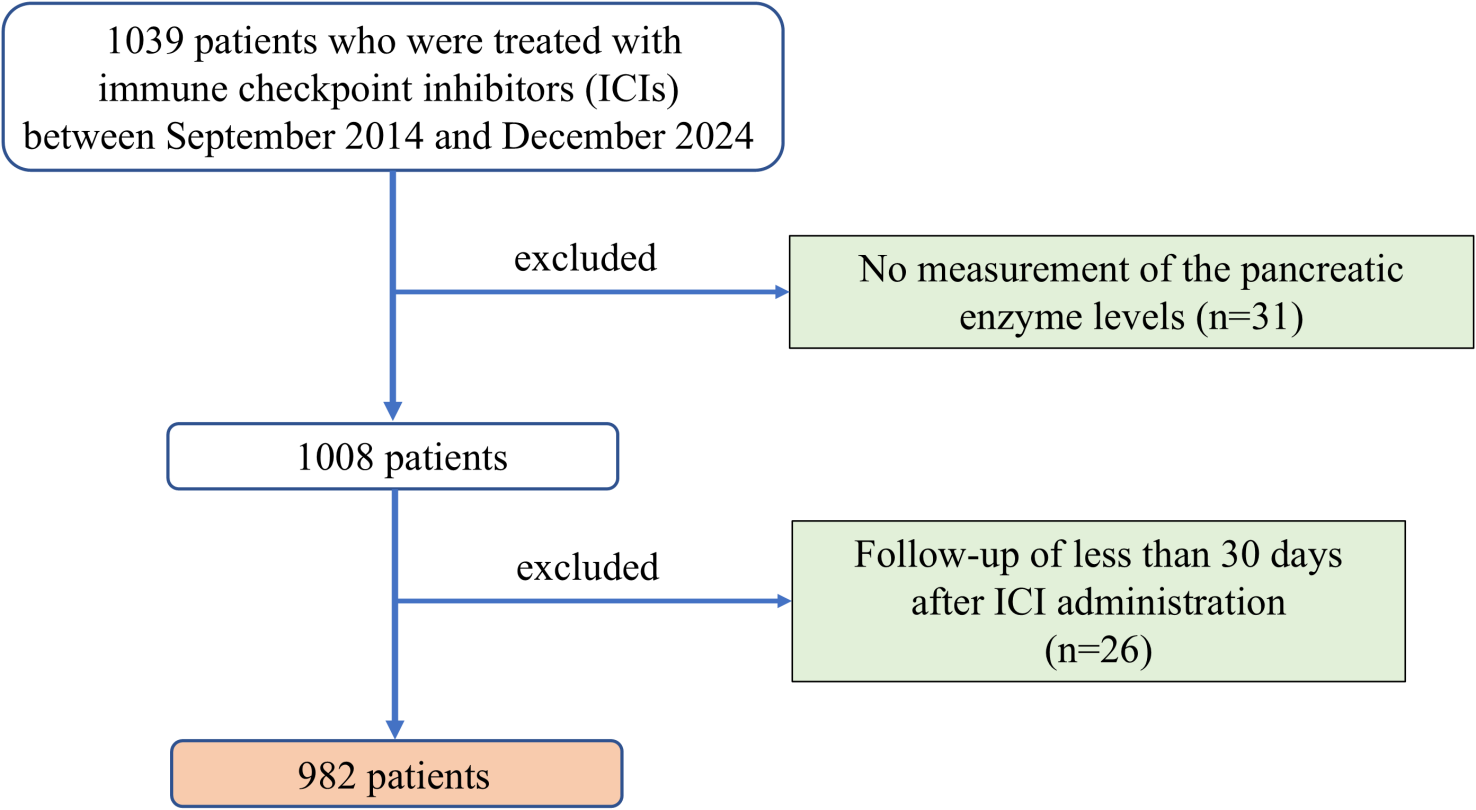
Diagram of the study design. A total of 1039 patients treated with ICIs between September 2014 and December 2024 were initially considered. Patients without pancreatic enzyme measurements (n=31) or with follow-up shorter than 30 days (n=26) were excluded. ICI, immune checkpoint inhibitor.

Herein, we retrospectively analyzed consecutive patients with malignant tumors who received ICI therapy at the University of Fukui. A diagram of the study is shown in **Figure 1**. A total of 1039 patients were enrolled between September 2014 and December 2024. Fifty-seven patients were excluded from this study, including 31 who had not undergone serum amylase measurements before and during ICI administration and 26 who had follow-up for less than 30 days after ICI administration. Ultimately, 982 patients who received ICI therapy were included in this study. This study was approved by the ethics committee of the University of Fukui (clinical research number: 20240112) and conducted in accordance with the Declaration of Helsinki. The requirement for written informed consent was waived because of the retrospective study design.

### 2.2 Data collection

The following clinical and oncological data of the enrolled patients were extracted from the electronic medical records: age, sex, type and clinical stage of primary cancer, autoimmune disease history, cytotoxic chemotherapy and ICI therapy history, type of ICI therapy including ICI combination therapy, and median ICI therapy sessions and duration. Moreover, routine blood tests, liver function, albumin, serum amylase, creatinine, and CRP levels were assessed before, during, and after ICI therapy. The primary cancer type was categorized as lung cancer, head and neck cancer, hepatocellular and bile duct cancer, gastric cancer, colon cancer, skin cancer, renal cancer, esophageal cancer, urothelial and bladder cancer, and other tumors. The clinical stage of primary cancer was classified according to the TNM classification of malignant tumors by the Union for International Cancer Control (UICC) system in use at that time. ICI therapy was categorized as ICI monotherapy, including PD-1, PD-L1, and CTLA-4 inhibitors; and ICI combination therapy, including PD-1/CTLA-4 and PD-L1/CTLA-4 therapies. ICI agents included ipilimumab, nivolumab, pembrolizumab, atezolizumab, durvalumab, avelumab, tremelimumab, and cemiplimab.

### 2.3 ICI-PI

In this study, we evaluated the peak levels of serum amylase, a pancreatic enzyme, and computed tomography (CT) findings for the pancreas during and after ICI therapy for all patients. ICI-PI was defined as ≥ Grade 2 elevation of serum amylase levels after ICI therapy initiation, according to the National Cancer Institute Common Terminology Criteria for Adverse Events (CTCAE) ver.5.0. We excluded patients with elevated serum amylase levels or acute pancreatitis attributed to other causes, such as chronic pancreatitis, alcohol, other non-ICI drugs, gallstones, pancreatic tumor, and endoscopic retrograde cholangiopancreatography. In addition, ICI-PI with pancreatitis was defined as satisfied two or more of the following criteria: 1) diagnosis of ICI-PI according to the above-mentioned definition; 2) presence of abdominal pain; and 3) imaging findings indicative of pancreatitis, which were further classified as acute pancreatitis-like (AP-like) and autoimmune pancreatitis-like (AIP-like) categories according to a previous study by Das, et al. (23). The diagnosis and grading of irAEs in any other organ, excluding ICI-PI, were based on CTCAE ver.5.0, and adverse events of ≥ Grade 2 were extracted.

### 2.4 Study outcome

The incidence and severity of ICI-PI were investigated, and risk factors for the development of ICI-PI, which could serve as predictive markers, were analyzed. Moreover, the association between ICI-PI development and long-term prognosis, including overall survival (OS), was evaluated. OS was defined as the time from ICI therapy initiation to death from any cause.

### 2.5 Statistical analysis

All statistical analyses were performed using R version 4.4.2 software (R Foundation for Statistical Computing, Vienna, Austria). Categorical variables are expressed as number of cases and frequency, and were compared using Fisher’s exact test. Continuous variables are described as the median and range. Univariate and multivariate analyses using binary logistic regression models were performed to evaluate risk factors for ICI-PI development by calculating the odds ratios (ORs) and 95% confidence intervals (CIs). OS was evaluated using Kaplan–Meier curves and log-rank tests to determine the differences between the groups. Because irAE is a time-varying factor, we performed landmark analysis to control immortal-time bias. In the landmark study design, patients with event before the preset time point, and those who experienced irAEs after this time were excluded. Considering previous studies, we set three time points: 3, 6, and 12 months. A *P*-value of < 0.05 was considered statistically significant.

## 3 Results

### 3.1 Baseline characteristics

The baseline characteristics of the 982 enrolled patients are shown in **Table 1**. The median age was 71.1 years (range, 9–94 years), with 28.4% (279/982) female patients. Regarding the primary cancer type, 30.7% (301/982), 11.8% (116/982), 10.9% (107/982), 0.7% (7/982), 10.9% (107/982), 9.0% (88/982), 8.0% (79/982), 6.1% (60/982), 5.8% (57/982), and 4.0% (39/982) had lung cancer, head and neck cancer, gastric cancer, colon cancer, hepatocellular and bile duct cancer, renal cancer, skin cancer, esophageal cancer, urothelial and bladder cancer, and breast and gynecological cancer, respectively. At the time of ICI administration, 75.2% (738/982) of patients had stage IV in the TNM classification, followed by stage III in 18.6% (182/982) and ¾ stage II in 6.3% (62/982). Moreover, 36 patients (3.7%) had an autoimmune disease history. In total, 637 patients (64.9%) had prior use of cytotoxic chemotherapy, and 61 (6.2%) had previously received ICI therapy. Considering the ICI therapy type, most patients were treated with PD-1 inhibitors (73.8%), followed by PD-L1 inhibitors (20.4%), and CTLA-4 inhibitors (0.8%). ICI combination therapy was administered in 5.0% (49/982) of patients. The median number of ICI therapy sessions was five, and the median duration of ICI therapy was 99.5 days. Laboratory findings at the first ICI administration are shown in **Table 1**. The median serum amylase level was 71.0 (range, 9–943) U/L. The median observation period was 274.5 days, and 438 patients (44.6%) died during the observation period.

**Table 1:** Baseline characteristics of the enrolled patients.

Characteristics	Total (n = 982)
Age, median, years	71.1 (9-94)
Female, no. (%)	279 (28.4)
Type of primary cancer, no. (%)	
Lung cancer	301 (30.7)
Head and neck cancer	116 (11.8)
Gastric cancer	107 (10.9)
Colon cancer	7 (0.7)
Hepatocellular and bile duct cancer	107 (10.9)
Renal cancer	88 (9.0)
Skin cancer	79 (8.0)
Esophageal cancer	60 (6.1)
Urothelial and bladder cancer	57 (5.8)
Breast and gynecological cancer	39 (4.0)
Others	21 (2.1)
TNM classification at the start of ICI therapy*, no. (%)	
I	7 (0.7)
II	55 (5.6)
III	182 (18.6)
IV	738 (75.2)
History of autoimmune disease, no. (%)	36 (3.7)
History of cytotoxic chemotherapy, no. (%)	637 (64.9)
History of ICI therapy, no. (%)	61 (6.2)
Type of ICI therapy, no. (%)	
ICI monotherapy	
PD-1 inhibitor	725 (73.8)
PD-L1 inhibitor	200 (20.4)
CTLA-4 inhibitor	8 (0.8)
ICI combination therapy	
PD-1 and CTLA-4 therapy	38 (3.9)
PD-L1 and CTLA-4 therapy	11 (1.1)
Number of ICI therapy, median, times (range)	5.0 (1-155)
Duration of ICI therapy, median, days (range)	99.5 (1-2444)
Hematological examination before ICI therapy	
Leukocyte, median (range), ×10^3^/uL	5.9 (0.4-37.2)
Neutrophil, median (range), ×10^3^/uL	3.8 (0.3-31.6)
Lymphocyte, median (range), ×10^3^/uL	1.1 (0.1-3.7)
NLR, median (range)	3.5 (0.5-95.0)
Platelet, median (range), ×10^4^/uL	23.2 (1.4-83.7)
Albumin, median (range), g/dL	3.5 (1.0-4.9)
Aspartate aminotransferase, median (range), U/L	21.0 (7-320)
Alanine aminotransferase, median (range), U/L	16.0 (1-330)
Alkaline Phosphatase, median (range), U/L	129.5 (17-2369)
Amylase, median (range), U/L	71.0 (9-943)
Creatinine, median (range), mg/dL	0.8 (0.2-11.4)
CRP, median (range), mg/dL	0.6 (0.0-26.9)
Observation period, median, days (range)	274.5 (30-3578)
Mortality outcome, no. (%)	438 (44.6)

CRP, C-reactive protein; CTLA-4, cytotoxic T-lymphocyte associated protein 4; ICI, immune checkpoint inhibitor; NLR, neutrophil to lymphocyte ratio; PD-1, programmed cell death 1; PD-L1, programmed cell death-ligand 1.

*The TNM classification was defined according to the TNM Classification of Malignant Tumors, 8th edition by the Union for International Cancer Control (UICC).

### 3.2 Incidence and clinical course of ICI-PI and pancreatitis in the enrolled patients

Among the 982 enrolled patients, 48 (4.9%) developed ICI-PI, with Grades 2, 3, and 4 in 41 (4.2%), 3 (0.3%), and 4 (0.4%) patients, respectively (**Table 2**). The median number of ICI administrations until ICI-PI onset was 3.0 (range, 1–50) times, and the median time from first ICI administration to ICI-PI onset was 96.0 days (range, 3–1576) days. Moreover, among the patients with ICI-PI onset, six (0.6%) developed pancreatitis. With regard to the imaging findings for ICI-related pancreatitis, four showed AP-like findings, while two showed AIP-like findings (known as type 3 AIP) (24, 25). Histological examination was performed using pancreatic tissue sampling by endoscopic ultrasonography-guided fine needle biopsy in two patients with ICI-related pancreatitis; marked lymphocytic infiltration, especially with CD8-positive T cells, was observed in the pancreatic tissue in both cases.

**Table 2:** Incidence of ICI-related pancreatic injury in the enrolled patients.

Variables	
ICI-related pancreatic injury (ICI-PI) *, no. (%)	
Grade 2, no. (%)	41 (4.2)
Grade 3, no. (%)	3 (0.3)
Grade 4, no. (%)	4 (0.4)
Total, no. (%)	48 (4.9)
ICI-PI without pancreatitis, *n* (%)	42 (4.3)
ICI-PI with pancreatitis, *n* (%)	6 (0.6)
Median number of ICI administration until onset of ICI-PI, times (range)	3.0 (1-50)
Median time from first ICI administration to onset of ICI-PI, days (range)	96.0 (3-1576)

ICI, immune checkpoint inhibitor; ICI-PI, ICI-related pancreatic injury.

*The grade of ICI-PI was defined according to the Common Terminology Criteria for Adverse Events (CTCAE) ver.5.0.

**Figure 2:**
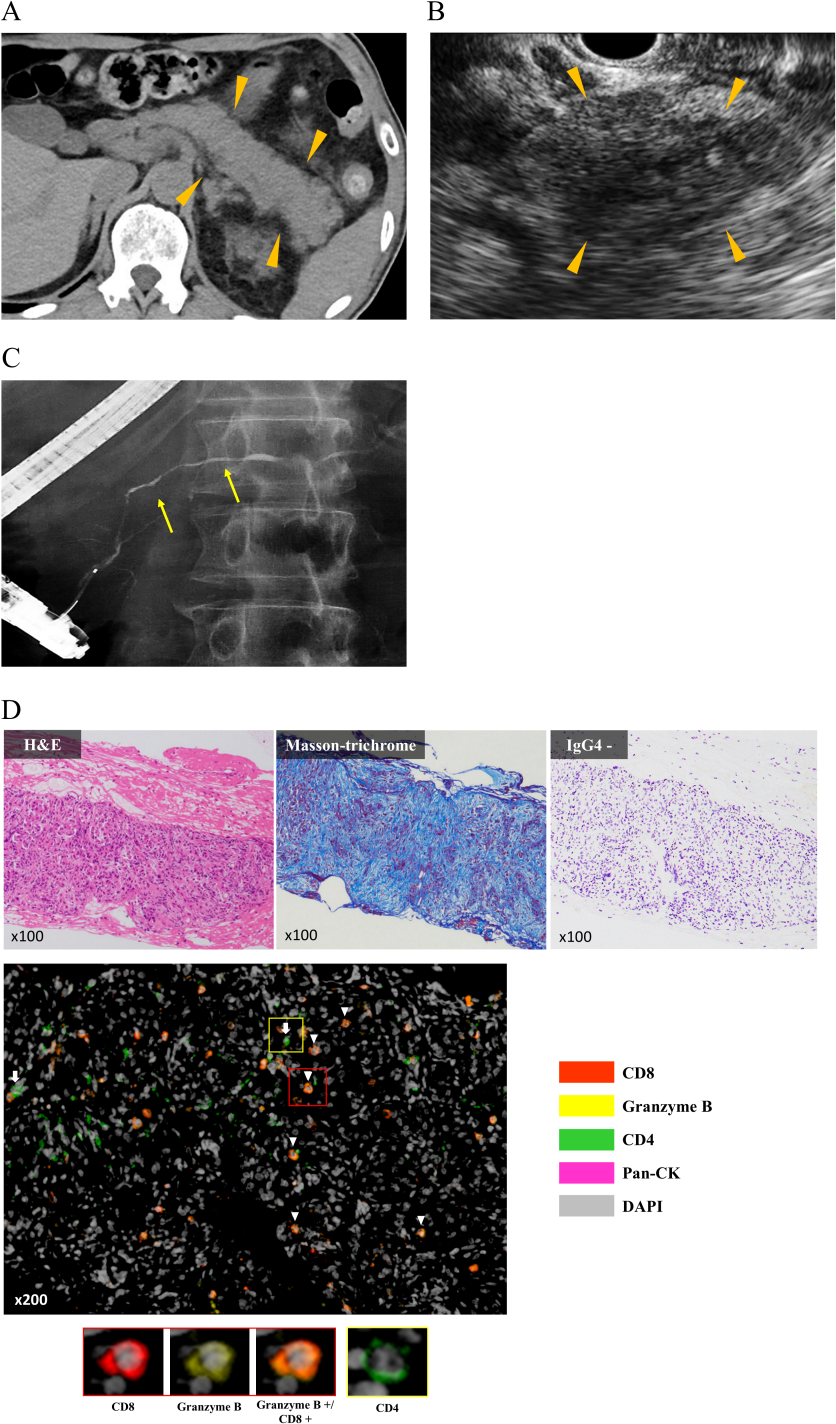
Characteristic clinical imaging and pathological findings in representative cases. **(A)** Abdominal computed tomography showing diffuse enlargement of the entire pancreas (arrow head). **(B)** Endoscopic ultrasonography showing hypoechoic and internal hyperechoic spots in the entire pancreas (arrow head). **(C)** Endoscopic retrograde pancreatography showing narrowing of the pancreatic duct (yellow arrow). **(D)** Histopathological analysis of endoscopic ultrasonography-guided fine needle biopsy specimens. H&E staining shows abundant lymphocytic infiltration into the pancreatic parenchyma, and Masson’s Trichrome staining shows severe fibrosis of the parenchyma. Single immunostaining shows negativity for IgG4. MFIH using a PerkinElmer Opal Kit shows that infiltrating lymphocytes into the pancreatic parenchyma were predominantly CD8 positive T cells that contained abundant granzyme B (arrow head) and a small number of CD4 positive T cells (white arrow). MFIH images were acquired using an automated multisector imaging system (Mantra version 2.0; ParkinElmer), and the patten of MFIH was composed of anti-CD4, anti-CD8, anti-Granzyme B, anti-pan Cytokeratin (pan CK), and anti-DAPI; opal 540 nm for anti-CD8, opal 570 nm for Granzyme B, opal 620 nm for anti-CD4, and opal 650 nm for anti-pan CK. CK, Cytokeratin; H&E, Hematoxylin and Eosin stain; MFIH, Multiplex fluorescence immunohistochemistry.

A representative case of ICI-related pancreatitis, including clinical imaging and pathological findings, is shown in **Figure 2**. The clinical imaging findings in this case were similar to those for typical AIP (**Figures 2A–C**). The histopathological analysis showed abundant lymphocytic infiltration into the pancreatic parenchyma and marked fibrosis of the pancreatic parenchyma (**Figure 2D**). Further, IgG4 staining was negative, which was different from the typical findings of type 1 AIP. In addition, the multiplex fluorescence immunohistochemistry revealed that the above infiltrating lymphocytes were predominantly CD8 positive T cells that contained abundant granzyme B and a small number of CD4 positive T cells (**Figure 2D**).

All six patients with ICI-related pancreatitis discontinued ICI therapy, and five received steroid therapy, which resulted in improvement in all patients. Only one patient with pancreatitis was rechallenged with the same type of ICI therapy following improvement in ICI-PI by steroid therapy, with subsequent relapse of ICI-related pancreatitis. On the other hand, in patients with ICI-PI without pancreatitis, 17 discontinued ICI therapy and 25 were carefully monitored while continuing ICI therapy, which resulted in improvement in all patients.

### 3.3 Risk factors for ICI-PI development

The results of univariate and multivariate analyses for risk factors related to ICI-PI using logistic regression models are shown in **Table 3**. In the univariate analysis, Number of ICI therapy ≥ 5 times (OR 2.01, 95% CI, 1.08–3.74; *P* = 0.029), Lymphocyte ≥ 1.0 ×10^3^/uL (OR 1.89, 95% CI, 1.03–3.46; *P* = 0.039), serum amylase level ≥ 70 U/L at the time of first ICI administration (OR 6.69, 95% CI 2.82–15.9; *P* < 0.001), and onset of ≥ Grade 2 other organ irAEs (OR 3.92, 95% CI, 2.13–7.19; *P* < 0.001) were identified as significant risk factors for ICI-PI development. Other clinical factors were poorly associated with ICI-PI development. In addition, the above factors with *P* < 0.05 in the univariate analysis were evaluated using multivariate analyses, which indicated that serum amylase levels ≥ 70 U/L at the time of first ICI administration (OR 6.10, 95% CI 2.55–14.6; *P* < 0.001) and onset of ≥ Grade 2 other organ irAEs (OR 3.49, 95% CI, 1.88–6.49; *P* < 0.001) were significant independent risk factors for ICI-PI development.

**Table 3:** Univariate and multivariate analyses of the risk factors for ICI-PI development in the enrolled patients.

Variables	Univariate analysis			Multivariate analysis		
	OR	95% CI	*P* value*	OR	95% CI	*P* value*
Age ≥ 70 years	1.29	0.71-2.34	0.398			
Female sex	1.20	0.62-2.34	0.591			
Stage IV in the TNM classification	1.72	0.93-3.13	0.085			
Type of primary carcinoma	1.14	0.96-1.35	0.125			
History of autoimmune disease	1.15	0.27-4.94	0.849			
History of cytotoxic chemotherapy	1.60	0.89-2.87	0.113			
History of ICI therapy	1.01	0.30-3.34	0.991			
PD-1 inhibitor therapy	1.26	0.60-2.64	0.545			
PD-L1 inhibitor therapy	1.20	0.57-2.51	0.636			
CTLA-4 inhibitor therapy	1.51	0.52-4.36	0.445			
ICI combination therapy	1.80	0.62-5.22	0.282			
Number of ICI therapy ≥ 5 times	**2.01**	**1.08-3.74**	**0.029**	1.50	0.78-2.87	0.215
Leukocyte ≥ 6.0 ×10^3^/uL	1.28	0.72-2.29	0.407			
Neutrophil ≥ 4.0 ×10^3^/uL	1.24	0.70-2.22	0.464			
Lymphocyte ≥ 1.0 ×10^3^/uL	**1.89**	**1.03-3.46**	**0.039**	1.64	0.88-3.06	0.119
NLR ≥ 5	1.08	0.60-1.93	0.800			
Platelet < 20.0 ×10^4^/uL	1.36	0.75-2.46	0.311			
Albumin < 3.5 g/dL	1.17	0.66-2.10	0.587			
Total bilirubin < 0.5 mg/dL	1.55	0.86-2.78	0.140			
Aspartate aminotransferase ≥ 20 U/L	1.20	0.66-2.16	0.552			
Alanine aminotransferase ≥ 20 U/L	1.48	0.75-2.94	0.262			
Alkaline Phosphatase ≥ 200 U/L	1.73	0.97-3.11	0.064			
Amylase ≥ 70 U/L	**6.69**	**2.82-15.9**	**< 0.001**	**6.10**	**2.55-14.6**	**< 0.001**
Creatinine ≥ 1.0 mg/dL	1.77	0.97-3.23	0.064			
CRP ≥ 1.0 mg/dL	1.02	0.56-1.85	0.942			
Onset of other organ irAE**	**3.92**	**2.13-7.19**	**< 0.001**	**3.49**	**1.88-6.49**	**< 0.001**

CRP, C-reactive protein; CTLA-4, cytotoxic T-lymphocyte associated protein 4; ICI, immune checkpoint inhibitor; ICI-PI, ICI-related pancreatic injury; irAEs, immune-related adverse events; NLR, neutrophil to lymphocyte ratio; PD-1, programmed cell death 1; PD-L1, programmed cell death-ligand 1.

*P value of less than 0.05 was considered statistically significant.

**Other organ irAEs were defined as irAEs with Grade 2 or higher, excluding ICI-PI, according to the Common Terminology Criteria for Adverse Events (CTCAE) version 5.0.

Variables considered significant are in bold.

### 3.4 Association between ICI-PI development and other organ irAEs

**Figure 3:**
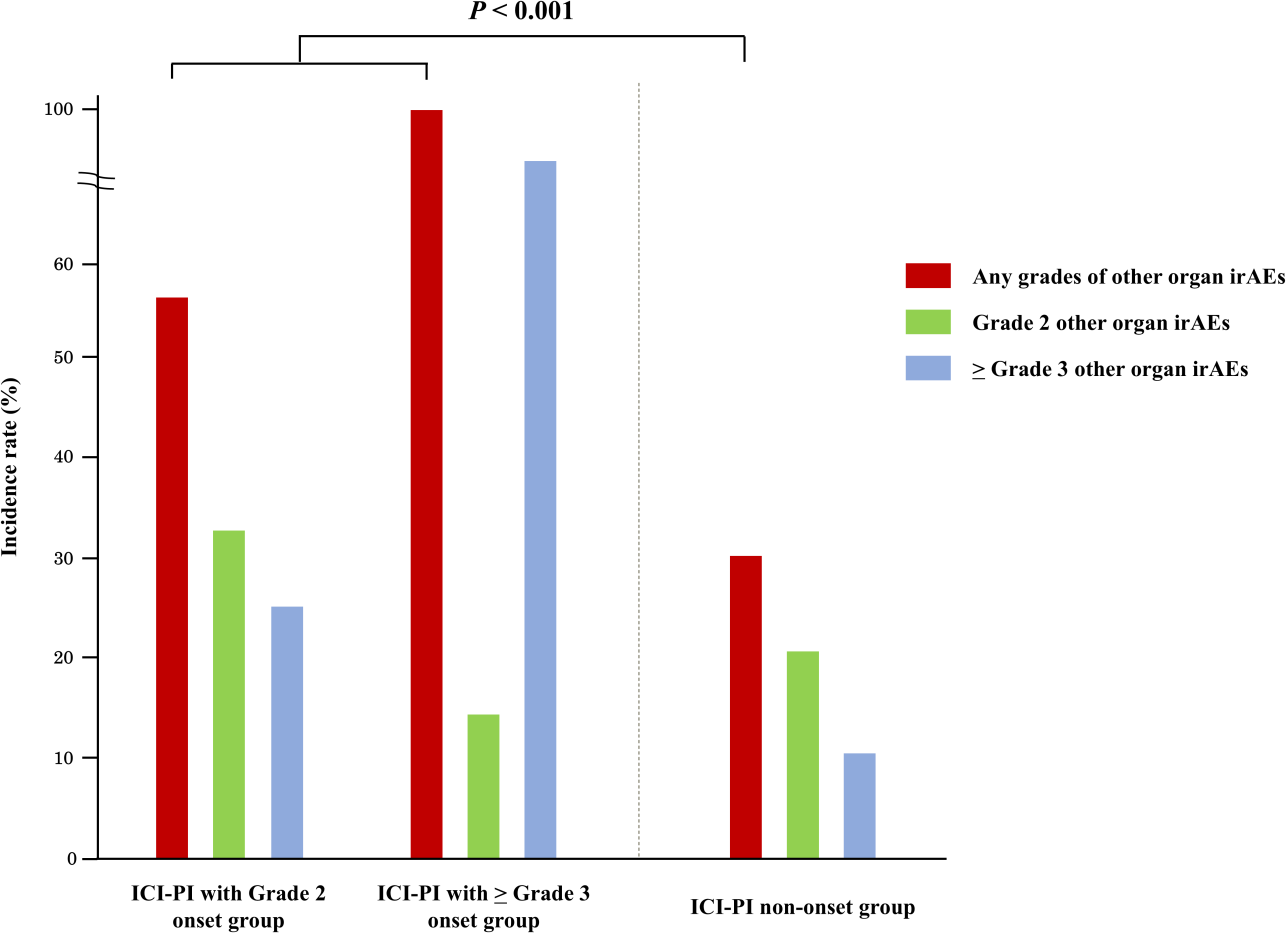
Association between the onset of ICI-PI and other organ irAEs. ICI-PI, ICI-induced pancreatic injury; irAEs, immune-related adverse events.

The incidence of organ irAEs, excluding ICI-PI, was 33.4% (328/982). The most common type of other organ irAE was hepatotoxicity, followed by hypothyroidism/thyroiditis, colitis/diarrhea, adrenal insufficiency, skin toxicity, and pneumonitis (**Table 4**). The association between ICI-PI development and other organ irAEs are shown in **Figure 3**. The incidence of other organ irAEs in the ICI-PI onset group, including the Grade 2 and ≥ Grade 3 onset groups, was significantly higher than that in the ICI-PI non-onset group (64.6% [31/48] vs. 31.8% [297/934]; *P* < 0.001). Moreover, the incidence of ≥ Grade 3 other organ irAEs was significantly higher in the ≥ Grade 3 ICI-PI onset group than in the Grade 2 ICI-PI onset and ICI-PI non-onset group (85.7% [6/7] vs. 26.8% [11/41] vs. 10.8% [101/934]; *P* < 0.001), indicating a positive correlation between the severity of ICI-PI and the severity of other organ irAEs. Among patients with ≥ Grade 3 ICI-PI development, the most common types of other organ irAEs included hypothyroidism/thyroiditis and adrenal insufficiency (**Table 5**). In patients with Grade 2 ICI-PI development, the most common type of other organ irAEs included hepatotoxicity and adrenal insufficiency (**Table 5**).

**Table 4:** Incidence of immune-related adverse events excluding ICI-PI in the enrolled patients.

Adverse events	Any grades*	Grades 3-5
Skin toxicity, n (%)	61 (6.2)	15 (1.5)
Hepatotoxicity, n (%)	89 (9.1)	40 (4.1)
Colitis/diarrhea, n (%)	66 (6.7)	24 (2.4)
Pneumonitis, n (%)	51 (5.2)	18 (1.8)
Nephrotoxicity, n (%)	13 (1.3)	6 (0.6)
Hypothyroidism/Thyroiditis, n (%)	75 (7.6)	2 (0.2)
Adrenal insufficiency, n (%)	65 (6.6)	14 (1.4)
Diabetes, n (%)	5 (0.5)	5 (0.5)
Musculoskeletal toxicity, n (%)	8 (0.8)	3 (0.3)
Myocarditis, n (%)	4 (0.4)	3 (0.3)
Peripheral neuropathy, n (%)	2 (0.2)	0 (0.0)
Uveitis, n (%)	2 (0.2)	0 (0.0)
Nausea/vomiting, n (%)	3 (0.3)	0 (0.0)
Infusion reaction, n (%)	5 (0.5)	2 (0.2)
Hematologic toxicity, n (%)	5 (0.5)	2 (0.2)
Gastritis, n (%)	1 (0.1)	1 (0.1)
Cholangitis, n (%)	1 (0.1)	1 (0.1)
Encephalopathy, n (%)	3 (0.3)	2 (0.2)
Otorhinolaryngological toxicity, n (%)	4 (0.4)	2 (0.2)
Others, n (%)	3 (0.3)	0 (0.1)

ICI, immune checkpoint inhibitor; ICI-PI, ICI-related pancreatic injury.

*Any grades of adverse events were defined as adverse events of Grade 2 or higher according to the Common Terminology Criteria for Adverse Events (CTCAE) version 5.0.

**Table 5:** Incidence of other organ immune-related adverse events in patients with ICI-PI.

Adverse events	Any grades*	Grades 3-5
Patients with ≥ Grade 3 ICI-PI (n=7)		
Skin toxicity, n (%)	1 (14.2)	0 (0.0)
Hepatotoxicity, n (%)	2 (28.4)	2 (28.4)
Hypothyroidism/Thyroiditis, n (%)	3 (42.6)	0 (0.0)
Adrenal insufficiency, n (%)	3 (42.6)	1 (14.2)
Colitis/diarrhea, n (%)	2 (28.4)	1 (14.2)
Diabetes, n (%)	1 (14.2)	1 (14.2)
Myocarditis, n (%)	1 (14.2)	1 (14.2)
Otorhinolaryngological toxicity, n (%)	1 (14.2)	1 (14.2)
Patients with Grade 2 ICI-PI (n=41)		
Skin toxicity, n (%)	5 (12.2)	1 (2.4)
Hepatotoxicity, n (%)	7 (17.1)	2 (4.9)
Cholangitis, n (%)	1 (2.4)	1 (2.4)
Colitis/diarrhea, n (%)	5 (12.2)	2 (4.9)
Pneumonitis, n (%)	6 (14.6)	2 (4.9)
Nephrotoxicity, n (%)	2 (4.9)	1 (2.4)
Hypothyroidism/Thyroiditis, n (%)	6 (15.0)	0 (0.0)
Adrenal insufficiency, n (%)	7 (17.1)	3 (7.3)

ICI, immune checkpoint inhibitor; ICI-PI, ICI-related pancreatic injury.

*Any grades of adverse events were defined as adverse events of Grade 2 or higher according to the Common Terminology Criteria for Adverse Events (CTCAE) version 5.0.

### 3.5 Impact of ICI-PI onset on the long-term prognosis of the enrolled patients

**Figure 4:**
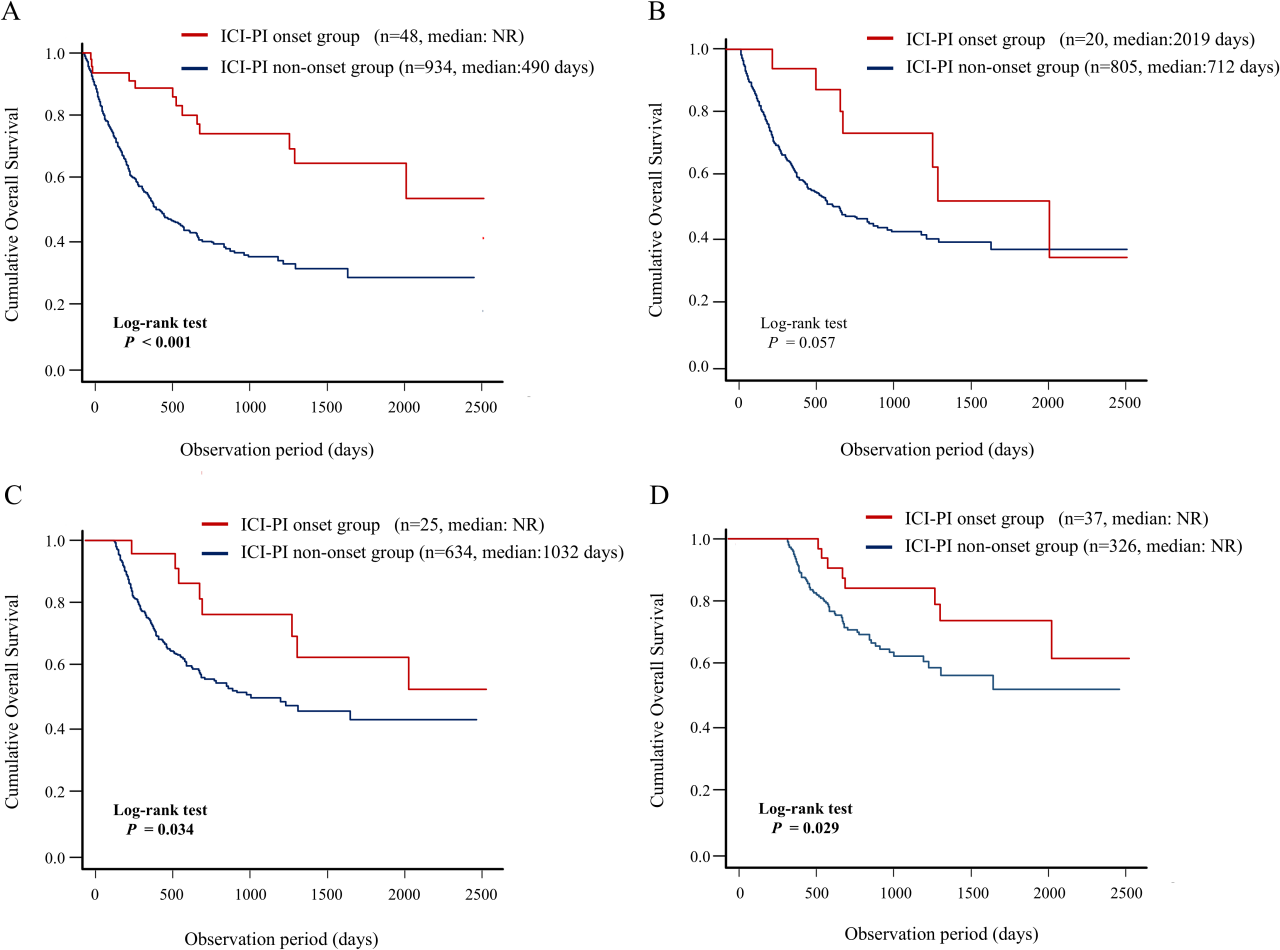
Impact of the onset of ICI-PI on the prognosis in enrolled patients receiving ICI therapy. Kaplan–Meier curve analysis for OS in patients with or without ICI-PI development. **(A)** OS under non-landmark analysis, **(B)** OS under 3-month landmark analysis, **(C)** OS under 6-month landmark analysis, and **(D)** OS under 12-month landmark analysis. ICI, immune checkpoint inhibitor; ICI-PI, ICI-induced pancreatic injury; irAEs, immune-related adverse events; NR, not reach; OS, overall survival.

In this study, we evaluated the association between ICI-PI onset and OS in the enrolled patients using the Kaplan–Meier curve analysis (**Figure 4**). The patients in the ICI-PI onset group (n = 48) had significantly better OS than those in the ICI-PI non-onset group (n = 934) (median days: not reached vs. 490 days; *P* < 0.001) (**Figure 4A**). The conditional survival rate in the ICI-PI onset and ICI-PI non-onset groups was 88.9% and 57.9% at 1 year survival, and 77.5% and 41.2% at 2 years survival, respectively.

In addition, considering the immortal-time bias, we performed landmark analysis (3-,6-, and 12-month) to analyze the relationship between irAEs and survival (**Figures 4B–D**). In the 6- and 12-month landmark analysis, patients who developed ICI-PI demonstrated significantly better OS than those who did not, consistent with the findings of the conventional analysis. These findings support the evidence of a close relationship between ICI-PI onset and better long-term prognosis.

### 3.6 Association between the number of organs with irAEs and long-term prognosis

**Figure 5:**
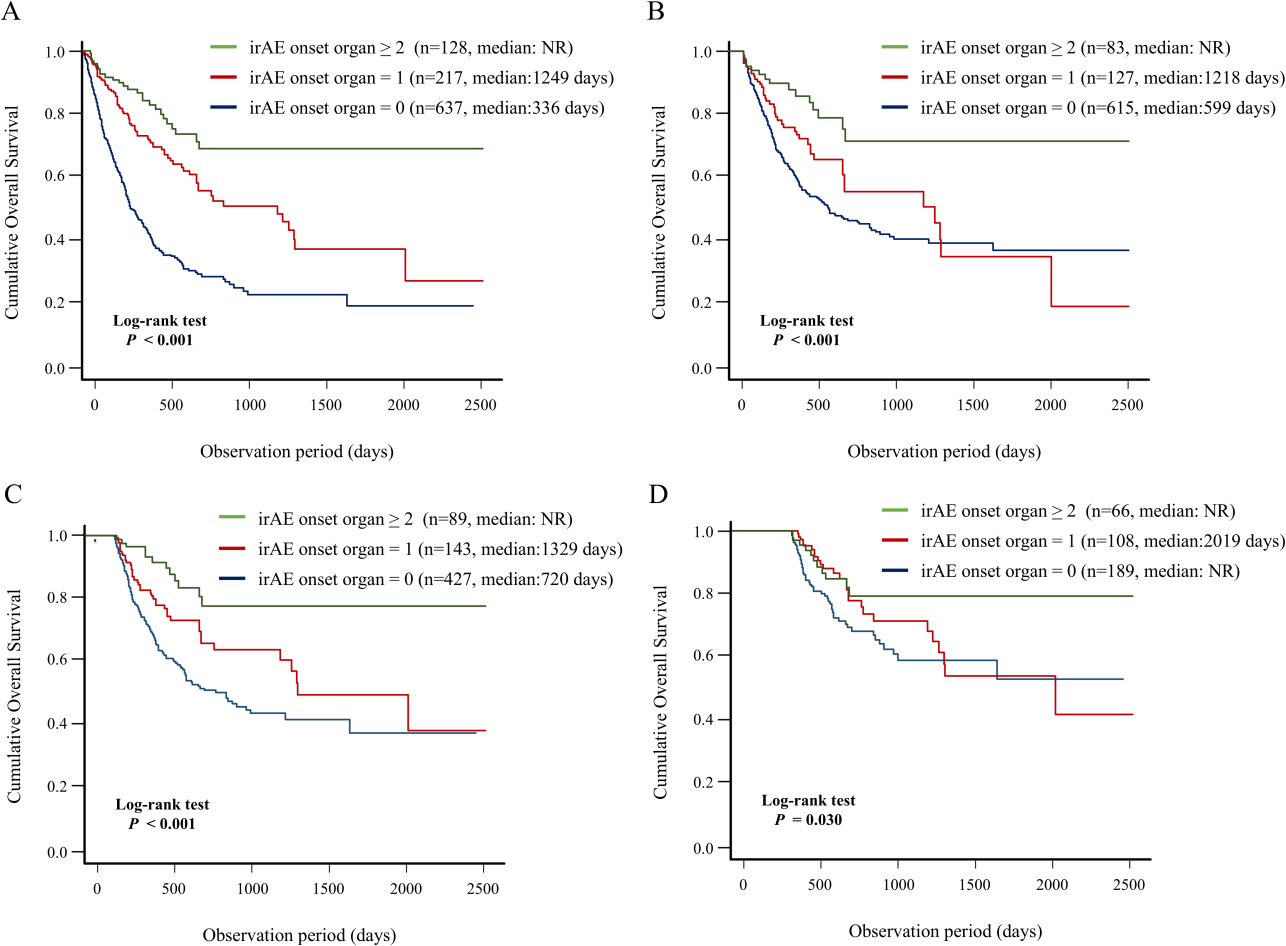
Association between number of organs with irAE onset and patient prognosis. Kaplan–Meier curve analysis for OS in the group of patients with irAE onset in two or more organs vs. the group of patients with irAE onset in a single organ vs. the group of patients without irAE onset. **(A)** OS under non-landmark analysis, **(B)** OS under 3-month landmark analysis, **(C)** OS under 6-month landmark analysis, and **(D)** OS under 12-month landmark analysis. irAEs, immune-related adverse events; NR, not reach; OS, overall survival.

Next, we evaluated the association between the number of organs with irAEs and OS using Kaplan–Meier curve analysis (**Figure 5**). The group of patients with irAE onset in the ≥ 2 organs had significantly better OS than did the other groups, including the groups of patients with irAE onset in a single organ and no irAE onset (median days: not reached vs. 1249 days vs. 336 days; *P* < 0.001) (**Figure 5A**). In addition, the results of landmark analysis (3-, 6-, and 12-month) were consistent with those of conventional analysis in all landmark subgroups (**Figures 5B–D**). These findings demonstrated a significant positive correlation between the number of organs with irAE and long-term prognosis.

## 4 Discussion

The present study found that high serum amylase levels at first ICI administration ​​and onset of other organ irAEs were potential novel risk factors for ICI-PI development in patients with advanced cancer receiving ICI therapy. In addition, we demonstrated that patients who developed ICI-PI had a better long-term prognosis than those who did not, which was corroborated by landmark analyses at three time points. To our knowledge, few studies, such as the recent multicenter study by Nagao et al. (9), have evaluated risk factors for ICI-PI development and the impact of ICI-PI on the long-term prognosis with such comprehensive detail as the present study.

The incidence of ICI-PI differed between several previous studies from the West and Japan (9, 11, 26). For example, Abu-Sbeih et al. demonstrated that 4% of patients who received ICI therapy developed ICI-PI (11). Further, one prior meta-analysis demonstrated asymptomatic lipase elevation in 2.7% patients following ICI use (26). More recently, Brandlmaier et al. reported a higher incidence of lipase elevation at 13.5% among melanoma patients receiving ICI therapy (27). In this study, the incidence of ICI-PI was 4.9% in the enrolled patients. These discrepancies in the ICI-PI incidence may be attributed to the varying definitions of ICI-PI in each report. In the present study, we defined ICI-PI as ≥ Grade 2 elevation of serum amylase levels following ICI therapy initiation according to CTCAE ver.5.0, and excluded any diseases that could cause elevated amylase levels. Moreover, the incidence of ICI-PI with pancreatitis has been reported to be approximately 0.3%–3.9% (9, 26, 28). The present study demonstrated that 0.6% enrolled patients developed ICI-related pancreatitis, which was consistent with the findings of previous studies. Although ICI-related pancreatitis is mild in most cases, and follows a favorable clinical course (26, 29), it can occasionally result in severe life-threatening conditions (30). Therefore, it is necessary to fully understand the clinical characteristics of ICI related pancreatitis for effective management of patients who receiving ICI therapy.

In recent years, several studies have identified risk factors for ICI-PI (10, 11, 26, 31). George et al. reported CTLA-4 inhibitors and malignant melanoma as high-risk factors for ICI-PI (26). Other previous studies demonstrated that ICI combination therapy causes a higher incidence of ICI-related lipase elevation (31). However, these factors were not identified as risk factors for ICI-PI in the present study, probably because of patient characteristics, such as race, primary cancer type, and type of ICI therapy. Moreover, we found that high serum amylase levels before ICI administration could be a novel risk factor for ICI-PI development. To our knowledge, this finding has not been previously reported. We speculated that in patients with abundant exocrine pancreatic function, activated T cell stimulation by ICI treatment may easily damage the pancreatic tissue. Furthermore, we demonstrated that the onset of other organ irAEs could be a novel risk factor for ICI-PI development. Previously, Jennings et al. showed that patients who developed irAEs in other organs were at an increased risk of developing immune-related hepatotoxicity (32). We further demonstrated that the onset of ICI-PI is closely related to the onset of irAEs in other organs, with a positive correlation between the severity of ICI-PI and that of other organ irAEs. Thus, thorough systemic management involving other organs may be important for the early detection and appropriate therapeutic intervention for ICI-PI.

Few studies have previously evaluated the association between ICI-PI and long-term prognosis. Nagao et al. revealed no significant difference in OS between patients with and without ICI-PI (9). On the other hand, our study demonstrated that patients who developed ICI-PI had a significantly better OS than did those without ICI-PI. Therefore, we considered that further detailed analysis is warranted to resolve this discrepancy. The occurrence of irAEs is time dependent, as patients who died before irAE development were considered to have no irAEs in the conventional analysis (33). This lead-time bias may result in overestimation of the impact of irAEs on prognosis (34, 35). To address this issue, we performed landmark analysis excluding patients who reached OS prior to the pre-specified time point to evaluate the association between ICI-PI onset and long-term prognosis. The results corroborated the association between ICI-PI onset and better prognosis. Large-scale studies are warranted to further consolidate our findings.

**Figure 6:**
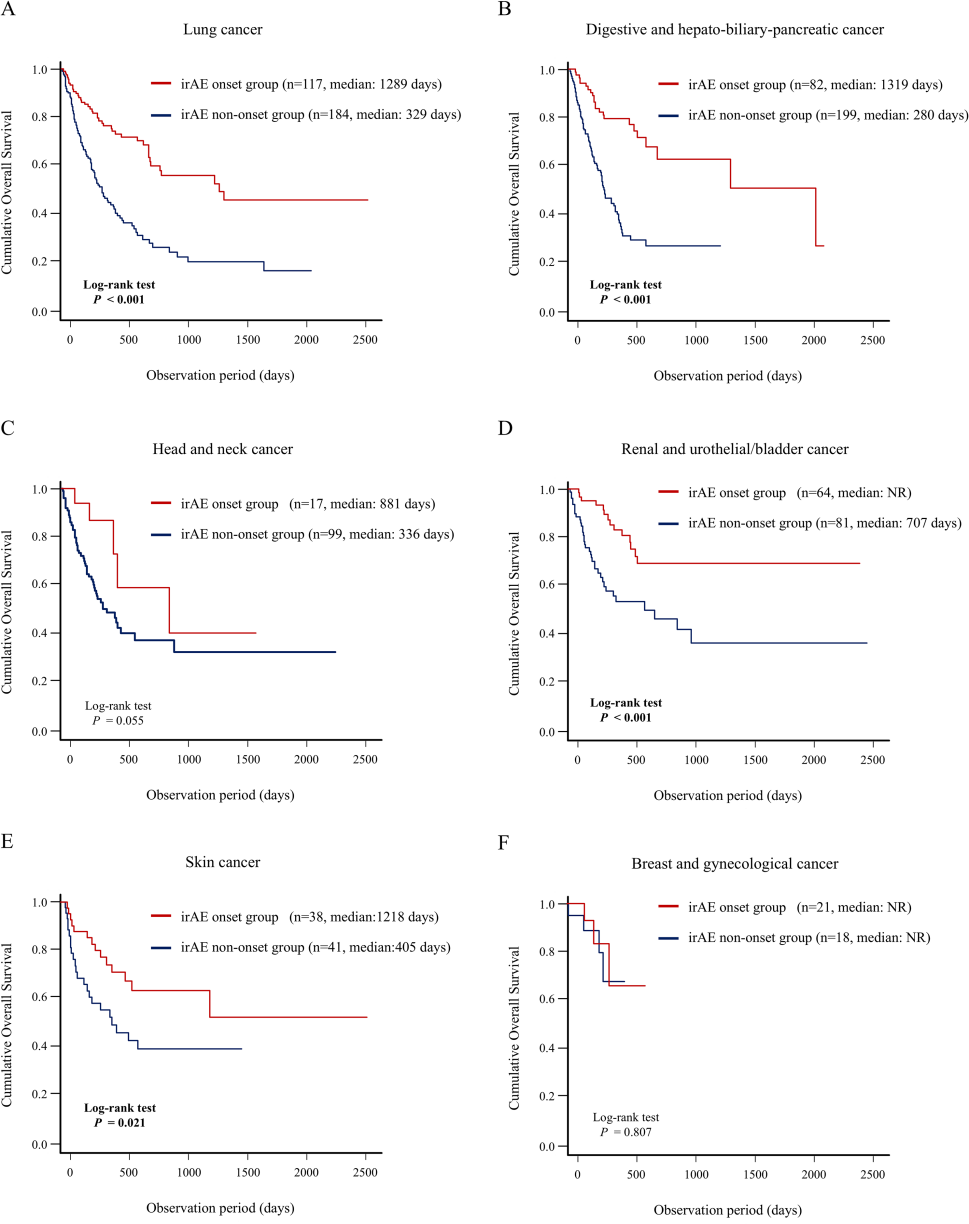
Association between irAE onset and patient prognosis according to type of the primary cancer. Comparison of the OS between the irAE onset group and irAE non-onset group using Kaplan–Meier curve analysis in the patients with **(A)** lung cancer, **(B)** digestive and hepatobiliary-pancreatic cancer, **(C)** head and neck cancer, **(D)** renal and urothelial/bladder cancer, and **(E)** melanoma, **(F)** breast and gynecological cancer. irAEs, immune-related adverse events; NR, not reach; OS, overall survival.

**Figure 7:**
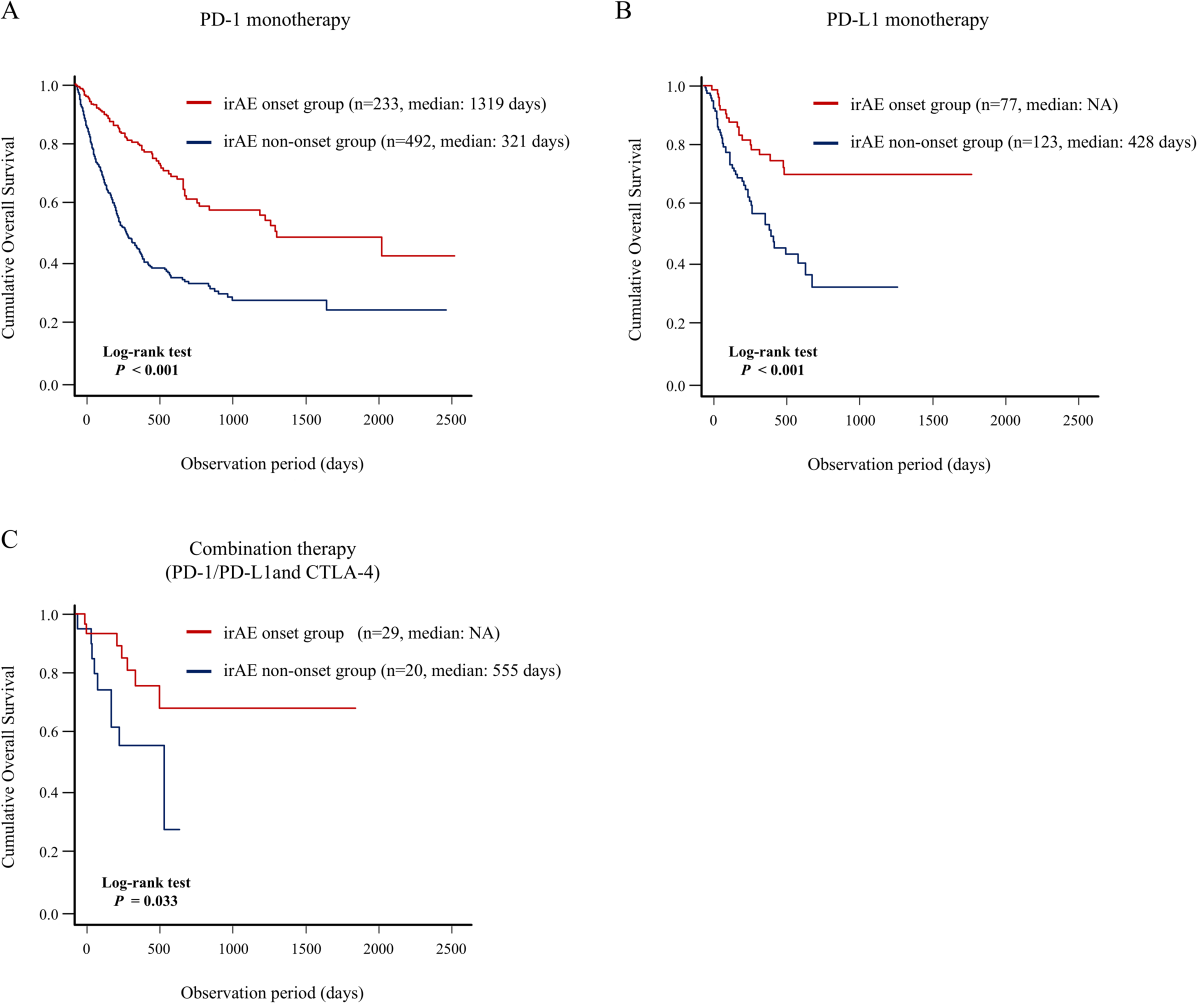
Association between irAE onset and patient prognosis according to type of ICI therapy. Comparison of the OS between the irAE onset group and irAE non-onset group using Kaplan–Meier curve analysis in the patients with **(A)** PD-1 monotherapy, **(B)** PD-L1 monotherapy, **(C)** PD-1/PD-L1 and CTLA-4 combination therapy. CTLA-4, cytotoxic T-lymphocyte antigen-4; irAEs, immune-related adverse events; NR, not reach; OS, overall survival; PD-1, programmed cell death 1; PD-L1, programmed cell death-ligand 1.

Furthermore, we demonstrated that patients who developed irAEs in multiple organs had a better prognosis, indicating a positive correlation between the number of organs with irAE and long-term prognosis. Thus, these patients could have greater therapeutic effect and higher irAE risk; they may share common pathobiology, including human leucocyte antigen genotypes or autoantibody formation (36, 37). Previously, Shankar et al. identified an association between multisystem irAEs and improved survival in patients with non-small cell lung cancer, which was consistent with our findings (38). Moreover, our landmark analysis strengthened the credibility of the finding that the onset of multiple organ irAEs could be closely associated with a favorable prognosis. We also analyzed the association between irAE development in any organ and long-term prognosis according to the primary cancer type (**Figure 6**) and to the type of ICI therapy (**Figure 7**). In detail, this study indicated that irAE development in any organ was significantly associated with better prognosis of patients with lung cancer, digestive and hepatobiliary-pancreatic cancer, renal and urothelial/bladder cancer, and skin cancer, and patients with PD-1 monotherapy, PD-L1 monotherapy, PD-1/PD-L1 and CTLA-4 combination therapy. These findings suggest that understanding irAE development in any organ may stratify the prognosis of patients receiving ICI therapy, thereby contributing to subsequent treatment strategies.

This study has several limitations. First, the retrospective study design may have contributed to selection and information bias. Notably, because this was a retrospective study, we were unable to perform a formal sensitivity analysis to exclude asymptomatic cases with isolated amylase elevation. However, to improve diagnostic specificity, we carefully excluded other potential causes of pancreatic enzyme elevation based on clinical presentation and imaging findings. Second, the external validity of this study was low because it was conducted in a single-center institution. In particular, in this study, data on prior treatments such as radiotherapy and targeted therapy against specific genomic alterations were not available, and their potential confounding effects could not be assessed, which may also influence the development of pancreatic injury. Also, key clinical variables, including tumor burden, PD-L1 expression status, genomic aberrations, and the use of corticosteroids or anti-inflammatory drugs, were not consistently available and thus could not be evaluated. These factors may affect both the efficacy of ICIs and the development of irAEs. In addition, the findings regarding the mechanism of ICI-associated pancreatic injury were based on only two biopsy cases, limiting the generalizability of the histological conclusions. Further studies with a larger number of biopsy-confirmed cases are needed to validate these observations. We hope that multicenter prospective studies should confirm the interesting findings of this study, including novel risk factors for ICI-PI development and the impact of ICI-PI on favorable prognosis.

In conclusion, our study identified high serum amylase levels before ICI administration and the development of other organ irAEs as novel risk factors for ICI-PI onset, and revealed that the long-term prognosis was better in patients with ICI-PI. These findings highlight the importance of thorough systemic management, including proactive evaluation of serum amylase levels and comprehensive monitoring for various irAEs, which can contribute to the early detection of ICI-PI and potentially lead to improved patient outcomes.

## Data Availability

The raw data supporting the conclusions of this article will be made available by the authors, without undue reservation.
